# Insecticides Resistance Status of* An. gambiae* in Areas of Varying Agrochemical Use in Côte D'Ivoire

**DOI:** 10.1155/2018/2874160

**Published:** 2018-10-08

**Authors:** Behi K. Fodjo, Benjamin G. Koudou, Emmanuel Tia, Jasmina Saric, Prisca B. N'dri, Marius G. Zoh, Christabelle S. Gba, Alida Kropf, Nestor B. Kesse, Mouhamadou S. Chouaïbou

**Affiliations:** ^1^Centre Suisse de Recherche Scientifique en Côte d'Ivoire (CSRS), Abidjan, Côte d'Ivoire; ^2^Université Nangui Abrogoua, Abidjan, Côte d'Ivoire; ^3^Liverpool School of Tropical Medicine, Liverpool, UK; ^4^Centre d'Entomologie Médicale et Vétérinaire, Université Alassane Ouattara, Bouaké, Côte d'Ivoire; ^5^Swiss Tropical and Public Health Institute, P.O. Box, CH-4002, Basel, Switzerland; ^6^University of Basel, P.O. Box, CH-4003, Basel, Switzerland; ^7^Université de Montpellier, Montpellier, France

## Abstract

**Background:**

Insecticide resistance monitoring of the malaria vectors to different classes of insecticides is necessary for resistance management. Malaria vector control management approaches are essentially based on IRS and LLINs. However, insecticide resistance is caused by several sources of selection and in case the selection pressure is from agricultural practices, then measures need to be taken to avoid a failure of the control methods put in place. The current study was undertaken to monitor the susceptibility of vectors to different classes of insecticides in areas of varying agrochemical use patterns.

**Methods:**

A survey to determine the agricultural chemical use pattern was undertaken in ten localities across Côte d'Ivoire. In addition, WHO susceptibility tests were carried out on adults* Anopheles gambiae s.l.* mosquitoes emerging from collected larvae from the sites surveyed. Four insecticides from each class of the four classes of insecticides were evaluated using the standard susceptibility test methods. Furthermore, the target site mutations involved in resistance mechanisms were identified following the Taqman assay protocols and mosquito species were identified using SINE-PCR.

**Results:**

The mortalities of all the* An. gambiae s.l* populations were similar regardless of the pesticide use pattern. The vectors were resistant to DDT, deltamethrin, and bendiocarb in all localities. In contrast, mosquitoes showed high susceptibility to malathion. High frequency of the Kdr-West gene allele was observed (70-100%). A single Kdr-East mutation was identified in a mosquito that harboured both Ace-1 and Kdr-West genes.

**Conclusion:**

Cultivated marshlands representing good habitats for mosquito development may deeply contribute to the selection of resistance genes given the intensive use of agrochemical for crop protection. In view of these, special attention must be given to them to mitigate mosquito resistance to insecticides.

## 1. Background

Malaria continues to challenge the various control strategies that have been deployed over several decades in endemic areas. The persistence of this disease is linked to several factors, wit, the most important ones being the absence of a vaccine, resistance of the parasite to antimalarial drugs, and resistance of vectors to insecticides [1–3]. Vector control remains one of the main pillars of malaria control and has contributed to the reduction of the spread of malaria in the absence of specific drug prophylaxis [4, 5]. Unfortunately, the efficacy of long lasting insecticidal nets (LLINs) and indoor residual spraying (IRS), which constitute the main tools of vector control, is being threatened by the resistance of the vectors to insecticides [5–10]. The resistance of vectors to several families of insecticides is highly widespread and has reached an extensive level geographically [2, 11, 12]. The management of those resistances is becoming a challenge for malaria control programmes worldwide. The World Health Organization (WHO)* Global Plan for Insecticide Resistance Management* (GPIRM) has proposed management approaches that are essentially based on IRS and LLINs [5]. Following those approaches, IRS and LLNIs were supposed to be the main sources of insecticide resistance of malaria vectors. However, if resistance selection pressure is originated from other sources, such as agriculture, then the methods proposed by the GPIRM may be much less effective. Indeed, the swamps used for rice and vegetable cultivation in the tropics offer ideal breeding sites for mosquitoes particularly* An. gambiae,* the main malaria vector [4, 13–18]. Some of the agricultural pesticides used in those swamps contain the same chemical active ingredients like the insecticides used for public health purpose [19–22]. Thus, the impact of agricultural pesticide usage has been highly indexed as a source of resistance to insecticides [22–27]. Exposure to different pesticides may lead to varied responses and mutations on the mosquito for better adaptation to the environment. So, for the fact that certain agrochemicals are potent inducers of genes involved in resistance development, the use of insecticidal treated tools for vector control in such areas may be less effective.

Several mechanisms due to mutations are involved in the resistance of malaria vectors to insecticide. The knock down resistance (kdr) mutation gene caused by the substitution of leucine to phenylalanine (L1014F) used to be specific to vectors in West Africa, while the substitution of leucine to serine (L1014S) was observed mostly in East Africa [28–31]. However, this clear separation has become increasingly blurred in recent years with the kdr-East gene being identified in Benin and Côte d'Ivoire in West Africa [32, 33] and the kdr-West gene being found in East African vectors [34].

Increasing monitoring of insecticide resistance is needed to guide mitigation and management strategies for vector control. When high level of insecticide resistance occurs, urgent action needs to be taken and guided by knowledge of the mechanisms involved in resistance. According to WHO recommendations, vector control interventions should be based on local entomological data including the vector composition and its susceptibility to insecticides [35]. Along the same line, this study is part of the insecticide resistance monitoring framework and aims to assess insecticide susceptibility and resistance mechanisms of* An. gambiae s.l.* collected from cultivated lowlands exposed to varying agricultural pesticides.

## 2. Methods

### 2.1. Study Sites

The study was carried out in 10 localities (i.e., Abengourou, Agboville, Azaguié, Dabou, MBé, San-Pédro, Tiassalé, Tiébissou, Toumodi, and Yamoussoukro) across different regions of Côte d'Ivoire. The practice of agriculture is common to all settings surveyed. Abengourou is located in the Eastern region of Côte d'Ivoire (6°43′N and 3°29′W). Coffee, cocoa, rubber, and palm oil are the main cash crops in Abengourou, while vegetable, rice, and banana are the main food crops in the area. The locality of Agboville is situated in the forest zone, in the south of Côte d'Ivoire (5°55′N and 4°13′W), with cultivation of coffee and cocoa being the main agricultural activity of this region. Food crops are dominated by banana, cassava, maize, yam, and rice. Azaguié is a city located in the forest zone in the south of Côte d'Ivoire (5°38′N and 4°05′W) and the main practiced agricultural activities practiced are cocoa, coffee, and food crops (e.g., cereals, rice, cassava, yams, and bananas). The locality of Dabou is situated in the same forest area (5°19′N and 4°23′W) with coffee, cocoa, rubber, palm oil, banana, and cassava being the main agricultural products grown. In the village Mbé at 8°6′N and 6°0′W, rice cultivation is the main economic activity of the population with 700 hectares of land devoted to rice cultivation in the area. The town of San-Pédro is located along the Atlantic Ocean at the gulf of Guinea (4°41′N and 6°39′W). San-Pédro is located within a dense forest area where perennial crops are dominant. Food crops include yams, potatoes, cassava, rice, corn, and smaller amounts of vegetables (e.g., eggplant, gumbo, spice, cabbage, cucumber, and lettuce). Tiassalé is located in the forest zone in the south of Côte d'Ivoire (5°53′N and 4°49′W) and relies on similar agricultural crops as Dabou. Tiébissou and Toumodi are located in central Côte d'Ivoire at 7°16′N and 5°29′W and 6°55′N and 5°03′W, respectively. Both settings have similar vegetation composed of wooded savannah and pre-forest savannah with gallery forests. The most dominant agricultural products in both settings are cereals, tubers, coffee, and cashew nuts. Yamoussoukro is also located in central Côte d'Ivoire (6°48′N and 5°17′W) with the cultivation of yams, cassava, and rice constituting the main agricultural activities.

The family of amino phosphonates, aryloxyacides, amide, and pyrimidine constituted herbicides class commonly used by farmers. Regarding insecticides, pyrethroids, organophosphates, organochlorines, carbamates, and neonicotinoid were commonly used.

LLINs are the main vector control tools used in these different localities.

### 2.2. Pesticide Use Survey

A survey on the use of pesticides by farmers was carried out in the ten localities using questionnaires. Between 78 and 106 farmers per locality were surveyed on their knowledge, attitude, and practices related to the use of pesticides in agriculture. Farmers were asked about the types of chemicals used, the doses by products, the frequency, and the rules of hygiene relating to the use of pesticides.

### 2.3. Susceptibility Test

The susceptibility of mosquitoes was assessed through the WHO cylinder test [36] and the mosquitoes used for the test were* An. gambiae s.l* wild strain species. Larvae were collected from the different site cultivated swamps.

After collection, the larvae of different areas were transferred and reared to adult stage in the insectary of the Centre Suisse de Recherches Scientifiques en Côte d'Ivoire (CSRS) at 27 ± 2°C and a relative humidity of 70%  ± 10%. After emergence, the adults were fed with cotton balls soaked in honey solution diluted to 10%. The susceptibility test performed according to the WHO protocol [36] involved exposure of 3–5-day old nonblood-fed female adults to a diagnostic dosage of the following insecticides: DDT (4%), deltamethrin (0.05%), bendiocarb (0.1%), and malathion (5%).* Anopheles gambiae* Kisumu strain was used as the reference susceptible strain and was tested simultaneously with the field populations. The mortality of the different tests achieved was interpreted according to the criteria proposed by WHO [36] as follows: mortality between 98% and 100% implies that the vectors are susceptible, mortality between 90% and 97% indicates the presence of resistance genes in the vector population which must be confirmed, and mortality less than 90% confirms the existence of resistance gene in the test population.

The resistance ratio (RR) of vectors to the various insecticides was determined from reports of the knock down time of 50% of the population (Kdt_50_) of wild mosquitoes and those of the susceptible Kisumu strain. This ratio expresses the level of resistance of the field strain compared with the susceptible* Kisumu* strain based on the knock down effect. The time at which 50% of the test population were knocked down (KDT_50_) was determined using PoloPlus software, via log-probit analysis.

### 2.4. Molecular Analysis of the Mosquito Vector

#### 2.4.1. DNA Extraction

Genomic DNA of the mosquitoes was extracted according to the method of Collins et al. [37]. In brief, whole mosquitoes were soaked in 2% cetyl trimethyl ammonium bromide (CTAB). The mosquitoes were crushed individually in 200 *μ*l of CTAB and incubated at 65°C for 5 min. A total of 200 *μ*l of chloroform were added and the resulting mixture was centrifuged for 5 min at 12,000 rpm. The supernatant was pipetted into a new 1.5 ml tube to which 200 *μ*l isopropanol was added; the mixture was centrifuged for 15 min at 12,000 rpm to precipitate the DNA. The supernatant was discarded subsequently and the DNA pellet formed at the bottom of tubes was purified with 70% ethanol. After a further centrifugation step at 12,000 rpm for 5 min, the ethanol was removed and the pellet dried on the bench over the night. The extracted DNA was reconstituted in 20 *μ*l DNase-free water (Sigma-Aldrich, United Kingdom) prior to storage at -20°C.

### 2.5. Identification of Anopheles gambiae s.s

The different species of* An. gambiae* (*An. gambiae s.s*. and* An. coluzzii*) were determined according to the SINE-PCR method previously described [38]. The primer F6.1A of sequence 5′-TCGCCTTAGACCTTGCGTTA-3′ was used to determine* An. coluzzii* and the primer R6.1B of sequence 5′-CGCTTCAAGAATTCGAGATAC-3′ for* An. gambiae s.s.* The incubation took place in a thermocycler of LongGene® type (A200 Gradient Thermal cycler; LongGene Scientific Instruments Co., Ltd Hangzhou, P.R. China) according to the following programme: 94°C for 5 min, 94°C for 25 s, and 54°C for 30 s; 72°C for 1 min repeated 35 times; and a final step at 72°C for 10 min to terminate the reaction. An agarose gel was prepared at 1.5% in TBE (Tris/borate/EDTA) containing ethidium bromide at 10 mg/ml. The PCR product was loaded on gel and allowed to migrate under a voltage of 140 V for 1 h. The result was visualized with a UV illuminator (BioDoc-It™ Imaging System; Upland, CA, USA). The profile of the expected bands by species was 479 bp for* An. gambiae coluzzii and *249 bp for* An. gambiae* s.s.

### 2.6. Identification of Resistance Genes

The real time PCR was used to investigate the presence of insecticide resistance genes including kdr-East and West and Ace-1 [39]. The reaction was carried out in an Agilent Stratagene MX3005 qPCR thermocycler (Agilent Technologies, Santa Clara, CA, USA) for each gene in a final volume of 10 *μ*l containing SensiMix and the specific probe containing FAM and HEX fluorochromes. FAM was used to detect the mutant allele, while HEX detected the wild-type susceptible allele. The amplification conditions used were 10 min at 95°C, 40 cycles of 10 s at 95°C, and 45 s at 60°C. Genotypes were scored from dual colour scatter plots produced by the device after incubation.

## 3. Results

### 3.1. Frequency of Pesticide Usage

**Figure 1:**
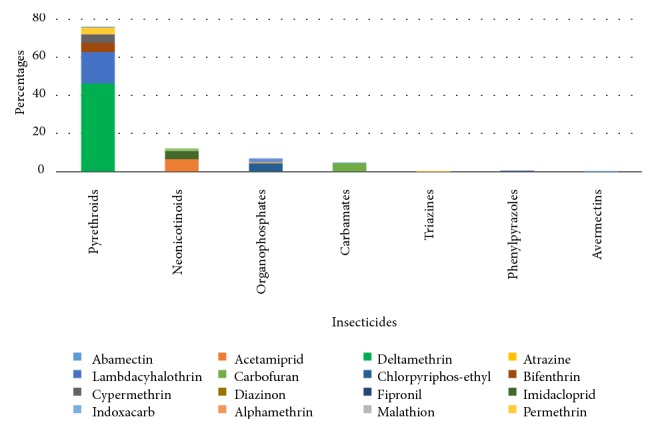
Proportion of different insecticide families used in the ten localities.

The results of pesticide usage surveys showed that 97.5% of the farmers surveyed used at least one pesticide. Three groups of pesticides were found in these ten localities. Among these pesticides, herbicides accounted for 61.9%, insecticides were 33.5% and fungicides 4.6%. The proportion of herbicides used in all localities was higher than that of insecticides except San-Pédro which is a cocoa growing area where insecticides accounted for 64.7% of the pesticides used. Among the insecticides, pyrethroids are the most commonly used (76. %) (Figure 1).

Pesticides were used at different frequencies depending on the crop areas. The low proportion of pesticides used have been observed in the area of culture of cashew, yams and cassava in the localities of Toumodi and Tiébissou while high proportion was observed in the rice fields and vegetable plots of Agboville, Tiassalé, and Dabou. The average number of pesticides treatment by farmer per year varied from 2.2 to 7.6 according the locality.

### 3.2. Susceptibility of Vectors to Insecticides

**Figure 2:**
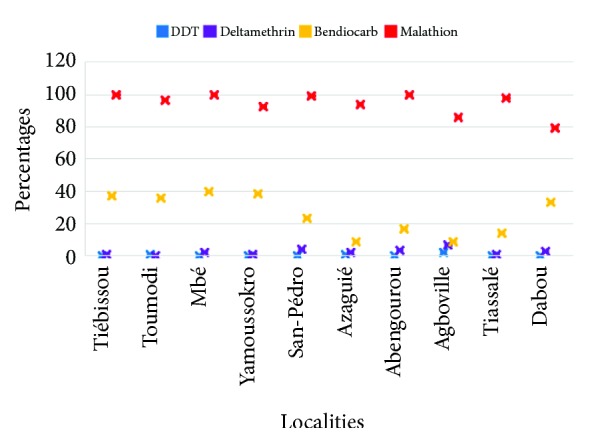
Level of resistance of* Anopheles gambiae* according to insecticide families in 10 localities in Côte d'Ivoire according to the intensity of agricultural pesticide use. The mortality generated by malathion remains higher than that of other insecticides in all localities, followed by bendiocarb, deltamethrin, and DDT.

Impregnated papers used for susceptibility testing resulted in 100% mortality of the susceptible Kisumu strain indicating the good quality of treated papers. Furthermore, control tube mortalities were less than 5%, implying any Abbot's correction. According to the susceptibility tests conducted on wild* An. gambiae* mosquitoes, the mortalities generated by each insecticide showed variations between the localities. (Figure 2). The resistance to DDT and deltamethrin was very high at the various sites assessed. Thus, the highest mortality against DDT was 2.2% and was observed in the locality of Dabou while the highest mortality against deltamethrin was 4% as observed in San-Pedro. For bendiocarb all mortality rates recorded were below 40%. In contrast, malathion recorded 100% mortality in Abengourou, Mbé, and Tiébissou, 99% in San-Pédro, and 98% in Tiassalé. In Toumodi and Yamoussoukro the mortality caused by malathion was 96.7% and 92.3%, respectively while in Dabou, the vectors developed resistance to malathion with a mortality of 79.3%.

### 3.3. Resistance Ratio and Knock Down

The RR_50_ varied among localities (Table 1). The lowest RR_50_ of deltamethrin was 3.5 as observed in Dabou and Yamoussoukro. However, the RR_50_ for deltamethrin could not be determined in Tiebissou because no mosquito had been knocked down during the exposure time. The highest rate of mosquitoes knocked down by deltamethrin (26%) was observed in Dabou from the 18^th^ minute. The few mosquitoes knocked down by DDT (12%) were after 30 min and were observed at Azaguié. The RR_50_ of bendiocarb varied between 2.5 and 3.9. The highest rate of mosquitoes knocked down by bendiocarb was observed at Toumodi (53%). Concerning malathion, the RR_50_ varied between 1.5 and 2.4 with 100% of the mosquitoes of the localities Abengourou and San-Pédro being knocked down before the end of the exposure time and after the 23^rd^ minute. Malathion recorded 100% knock down rate after 18 minutes at Tiébissou and represented the fastest knock down effect.

### 3.4. Molecular Species

**Figure 3:**
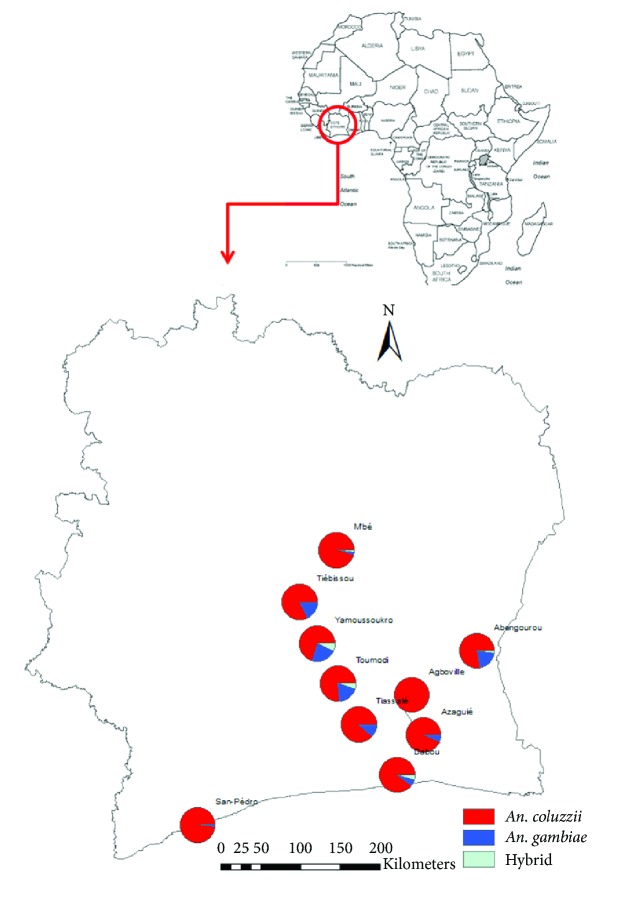
Proportion of different species by locality.

Among the 436* An. gambiae *specimens characterized,* An. coluzzii *was the most represented species with a proportion of 82.2% followed by* An. gambiae s.s. *with 16.1%. Nine mosquitoes were characterized as hybrids (2.1%). In the locality of Agboville all mosquito specimens analyzed were* An. coluzzii* (Figure 3).

### 3.5. Resistance Genes

The kdr-East, kdr-West, and Ace-1 genes were all identified in the vectors. Among these resistance genes, kdr-West was the most frequently expressed within the population of vectors with an allelic frequency ranged between 0.7 (Tiebissou) and 1 (Abengourou). All the samples tested had at least one resistant allele to the kdr-West gene. The proportion of homozygous individuals of this mutant allele was 71.9% in general while that of heterozygous individuals was 28.1%. As for the kdr-East gene, 98.5% of the genes expressed were of the susceptible genotype and only a single sample of the locality of Tiébissou was heterozygous for this gene. This same mosquito harboured the kdr-West gene and the Ace-1 gene at the heterozygous stage. Concerning the Ace-1 mutation, the allelic frequency of the mutant gene ranged between 0.07 and 0.5 with 0.014 for homozygotes and 0.54 for heterozygotes (Table 2).

## 4. Discussion

The susceptibility tests carried out on* An. gambiae* populations sampled in 10 different localities across Côte d'Ivoire highlight the extent of resistance of this vector to the four classes of insecticides commonly used for malaria vector control. The level of resistance developed against deltamethrin, DDT, and bendiocarb was found to be very high and in line with the trends reported by previous studies [11, 12, 40]. The strong resistance observed with high kdr allelic frequencies suggested a fixation of this resistance gene within the population of* An. gambiae* in Côte d'Ivoire. The mortalities generated among the* An. gambiae* population were similar regardless of the pesticide use patterns as high level of resistance where found in low moderate and high pesticide used area. A similar level of insecticide resistance across areas of different pesticide exposure-intensity may be explained by vector control activities in each of the areas assessed such as the distribution of LLINs by the national malaria control program (NMCP) since decade. A specific influence of agrochemicals to development of resistance of* An. gambiae* populations could not be dissociated in the current set up. However, we were unable to locate agricultural areas with complete absence of pesticide-use; therefore, the role that agrochemicals may play in fostering insecticide resistance should not be dismissed. Both the presence of hybrids and the migration of vectors can contribute to genetic shuffling and favour the homogenization of populations of resistant vectors. Large-scale aerial spraying of agricultural pesticides in plantations by agroindustrial firms that are widely established in Côte d'Ivoire should also be considered in future studies.

Although the use of DDT is prohibited in agriculture as well as for public health in Côte d'Ivoire, the current study revealed a high resistance of mosquitoes to this product. This high DDT resistance observed in the mosquito populations across the country may be attributed to both the strong residues of this product in the nature [41] having been used in the past and the cross-resistance between DDT and pyrethroids which were shown to account for more than 90% of chemical used by farmers [12].

In Tiébissou, a single* An. gambiae s.s*. mosquito was found to possess the kdr-West (L1014F), kdr-East (L1014S) and Ace-1 (G119S) mutations simultaneously. The co-occurrence of both the kdr mutations (L1014F/S) has been reported from West, Central and also East Africa [42–44]. However, this is the first time these three alleles associated with insecticide resistance were found in a single specimen in Côte d'Ivoire.

The presence of hybrids is a very serious threat to vector control as this can favour the spread of these traits. Hence, our research highlights the urgent need for a compound with a novel mode of action, in addition to those already existing, to eliminate such individuals within the vector population.

Although Ace-1 mutation can lead to resistance to both carbamates and organophosphates, the high resistance level to carbamates in one hand and low resistance to organophosphates in another hand found in the current study suggests that the resistance mechanism to these compounds involved other pathways and requires further investigations.

The overall situation of resistance as observed in the current study no longer allows dissociating the impact of public health insecticides to that of the agricultural pesticide usages. Yet this knowledge is key to evaluate the actual contribution of each domain to the selection of resistant individuals, owing to the strong focus on vector control in current malaria control programmes. In addition, until specific insecticides dedicated solely to vector control are available, combined management policies need to be established to deal with resistance of vectors to these same set of compounds used in agricultural and for public health purpose.

All the actors involved in vector control have unanimously agreed on the need of an adequate resistance management practices, yet most programme or project activities are limited to suggestions without practical field management actions. The proof of the extent of the resistance phenomenon must be conducive to raise awareness and training of farmers and users of public health insecticides for better management of insecticide resistance. To this end, ‘field school' projects to train farmers on the proper way to use the pesticides should be initiated in areas of intense cultivation.

In a context of resistance generalization, a debate on the usefulness of continued implementation of LLINs arises. Although LLINs provide personal protection, the strong levels of resistance observed with pyrethroids used for impregnating bed nets may put communities at risk owing to the increment of resistance selection. By eliminating susceptible individuals, the propagation of resistance genes may be intensified by the multiplication of resistant individuals. A reintroduction of no insecticide impregnated bed nets may be therefore preferable in such setting, providing personal protection in the same way as impregnated LLINs, but with the advantage of not selecting for mutant genes. The use of organophosphates in the current context in IRS may be a good alternative considering the susceptibility of the vectors to malathion. There is a real danger that resistance, if not monitored closely, may lead to a loss of all the gains made in recent decades in malaria control, notably the fall of the global burden of malaria by nearly 29% [45]. It is therefore of utmost importance to take appropriate and context-specific measures.

## 5. Conclusions

This study revealed strong resistance of malaria vectors to deltamethrin, DDT, and bendiocarb in 10 varying agrochemicals use areas in Côte d'Ivoire. Malathion induced high mortality in most localities but vector resistance against this insecticide observed in Dabou and Agboville may be the beginning of widespread resistance in the near future if no appropriate management measures are taken.

In view of the abundance of cultivated marshlands and their intense use of pesticides, special attention must be given to them to prevent the emergence of resistant mosquitoes.

The strong resistance to pyrethroids observed and the high frequency of resistance genes may hinder the effectiveness of mosquito nets. However, organophosphates appears to be a good alternative for vector control. The identification of individual mosquitoes possessing all three mutation-type resistance genes appears to be an additional threat to current vector control tools if this trait were to spread more widely and urges close surveillance.

## Figures and Tables

**Table 1:** Resistance ratio (RR_50_) of mosquito populations (*An. gambiae*) to deltamethrin, malathion, bendiocarb, and DDT. CI_50_: confidence interval at 50%; KdT_50_: knockdowntime of 50% of the population; *KdT*_*50*_ of the wild strain divided by KdT_50_ of the Kisumu reference strain; RR_50_: resistance ratio at 50%; /: cannot be determined (no knockdown).

**Insecticides by localities**	**Kdt**_**50 **_ **(CI**_**50**_ **) kisimu (min)**	**Kdt**_**50 **_ **(CI**_**50**_ **) wild strain (min)**	**RR**_**50**_
**Azaguié**			
**Deltamethrin (0.05**%**)**	31.9 (25.8–38.5)	114.3 (90.3–191.1)	3.6
**Malathion (5**%**)**	29.6 (28.4–30.9)	51,3 (48.7–54.6)	1.7
**Bendiocarb (0.1**%**)**	31.6 (30.5–32.6)	79,5 (72.5–93.3)	2.5
**DDT (4**%**)**	22.3 (21.7–22.9)	/	/
**Tiébissou**			
**Deltamethrin (0.05**%**)**	31.9 (25.8–38.5)	/	/
**Malathion (5**%**)**	31.6 (30.5–32.6)	46.0 (45.0–47.1)	1.5
**Bendiocarb (0.1**%**)**	18.7 (18.1–19.3)	56.5 (54.3–59.2)	3
**DDT (4**%**)**	22.3 (21.7–22.9)	/	/
**Abengourou**			
**Deltamethrin (0.05**%**)**	21.7 (20.7–22.6)	80.2 (72.9–92.2)	3.7
**Malathion (5**%**)**	31.4 (29.9–32.9)	33.7 (32.9–34.4)	1.1
**Bendiocarb (0.1**%**)**	28.2 (26.9–29.5)	37.6 (33.7–45.2)	2.9
**DDT (4**%**)**	22.3 (21.7–22.9)	/	/
**Agboville**			
**Deltamethrin (0.05**%**)**	21.7 (20.7–22.6)	99.32 (85.5–122.8)	4.6
**Malathion (5**%**)**	28.2 (26.9–29.5)	61.9 (57.8–67.8)	2
**Bendiocarb (0.1**%**)**	28.2 (26.9–29.5)	419.3 (224.7–1492.3)	14.9
**DDT (4**%**)**	22.3 (21.7–22.9)	/	/
**San-Pédro**			
**Deltamethrin (0.05**%**)**	21.7 (20.7–22.6)	83.45 (75.1–97.8)	3.9
**Malathion (5**%**)**	31.6 (30.5–32.6)	33.68 (32.9–34.,4)	1.1
**Bendiocarb (5**%**)**	25.5 (23.1–27.7)	37.6 (33.7–45.1)	1.5
**DDT (4**%**)**	22.3 (21.7–22.9)	/	/
**Yamoussoukro**			
**Deltamthrin (0.05**%**)**	31.9 (25.8–38.5)	111.9 (88.6–160.3)	3.5
**Malathion (5**%**)**	31.6 (30.5–32.6)	72.4 (67.9–78.0)	2.3
**Bendiocarb (0.1**%**)**	18.7 (18.1–19.3)	64.6 (61.4–68.7)	3.5
**DDT (4**%**)**	22.3 (21.7–22.9)	/	/
**Dabou**			
**Deltamethrin (0.05**%**)**	31.9 (25.8–38.5)	111.9 (88.6–160.3)	3.5
**Malathion (5**%**)**	31.6 (30.5–32.6)	66 (62.2–71.0)	2.1
**Bendiocarb (0.1**%**)**	18.7 (18.1–19.3)	72.4 (65.6–82.4)	3.9
**DDT (4**%**)**	22.3 (21.7–22.9)	/	/
**MBé**			
**Deltamethrin (0.05**%**)**	29.7 (29.8–30.5)	/	/
**Malathion (5**%**)**	31.6 (30.5–32.6)	72.9 (68.8–79.1)	2.3
**Bendiocarb (0.1**%**)**	18.7 (18.1–19.3)	70.3 (66.7–75.6)	3.8
**DDT (4**%**)**	22.3 (21.7–22.9)	/	/
**Tiassalé**			
**Deltamethrin (0.05**%**)**	21.7 (20.7–22.6)	220.4 (151.6–431.7)	10.2
**Malathion (5**%**)**	31.4 (29.9–32.9)	63.4 (60.5–66.9)	2
**Bendiocarb (0.1**%**)**	28.2 (26.9–29.5)	123.5 (94.8–227.4)	4.4
**DDT (4**%**)**	22.3 (21.7–22.9)	/	/
**Toumodi**			
**Deltamethrin (0.05**%**)**	31.9 (25.8–38.5)	299.2 (155.6–2521.4)	9.4
**Malathion (5**%**)**	31.6 (30.5–32.6)	55.4 (54.1–67.0)	1.8
**Bendiocarb (0.1**%**)**	18.7 (18.1–19.3)	50.7 (48.8–62.9)	2.7
**DDT (4**%**)**	22.3 (21.7–22.9)	/	/

**Table 2:** Allelic frequency of different mutational genes in malaria vectors in 10 localities in Côte d'Ivoire. The allelic frequency is the rate of presence of the resistance allele within the vector populations.

**Localities**	**West-Kdr Freq.(R)**	**East-Kdr Freq.(R)**	**Ace-1 Freq.(R)**
**Azaguié**	0.92	0	0.33
**Tiébissou**	0.7	0.03	0.13
**Toumodi**	0.8	0	0.33
**Mbé**	0.77	0	0.13
**Yamoussoukro**	0.96	0	0.32
**Abengourou**	1	0	0.20
**San-Pédro**	0.93	0	0.07
**Agboville**	0.79	0	0.5
**Tiassalé**	0.7	0	0.4
**Dabou**	0.9	0	0.43

## Data Availability

Data generated or analyzed during this study are included in this published article.

## Abbreviations

Ace-1: Insensitive acetylcholinesterase
CTAB: Cetyl trimethyl ammonium bromide
DDT: Dichlorodiphenyltrichloroethane
DNA: Deoxyribonucleic acid
GPIRM: Global Plan for Insecticide Resistance Management
IRS: Indoor residual spraying
Kdr: Knockdown resistance
KdT50: Knockdown time of 50%
LLINs: Long lasting insecticidal nets
PCR: Polymerase chain reaction
RR: Resistance ratio
WHO: World Health Organization.

## Supplementary Materials

Supplementary Materials The first 40 tables are the mosquito susceptibly test data sheets from Abengourou, Abidjan, Agboville, Azaguié, Dabou, M'bé, San-Pédro, Soubré, Tiassalé, Tiébissou, Toumodi, and Yamoussoukro localities to deltamethrin, bendiocarb, malathion, and DDT. Table 41 is the mutation data sheets. And the last table is the proportion of different insecticide families used in the ten localities.
