# Supplementary material for: Insecticides Resistance Status of An. gambiae in Areas of Varying Agrochemical Use in Côte D'Ivoire
Source: Biomed Res Int. 2018 Oct 8;2018:2874160. doi: 10.1155/2018/2874160 (PMC6196986; doi:10.1155/2018/2874160)
Supplement: Supplementary Materials — The first 40 tables are the mosquito susceptibly test data sheets from Abengourou, Abidjan, Agboville, Azaguié, Dabou, M'bé, San-Pédro, Soubré, Tiassalé, Tiébissou, Toumodi, and Yamoussoukro localities to deltamethrin, bendiocarb, malathion, and DDT. Table 41 is the mutation data sheets. And the last table is the proportion of different insecticide families used in the ten localities. [file 2874160.f1.pdf]

TEST DE SENSIBILITE DES VECTEURS DE PALUDISME AUX INSECTICIDES

**Localities**	**Abengourou**
**Date**	**5/16/2016**
**Espèce vectrice**	***An. gambiae***
**Insecticide testé**	**Deltamethrin 0,05%**
**Replicat**	**4**

	TEMPERATURE		HUMIDITE RELATIVE	
	AU DEBUT	A LA FIN	AU DEBUT	A LA FIN
PERIODE D'EXPOSITION	25,2°C	24,9°C	53%	54%
PERIODE D'OBSERVATION				

	Tube 1	Tube 2	Tube 3	Tube 4	Total Test	Témoin 1
Nombre de moustiques exposés	25	25	24	23	97	25
Nombre de moustiques Kd après :						
5 mn	0	0	0	0	0	0
8 mn	0	0	0	0	0	0
10 mn	0	0	0	0	0	0
13 mn	0	0	0	0	0	0
15 mn	0	0	0	0	0	0
18 mn	0	0	0	0	0	0
20 mn	0	0	0	0	0	0
23 mn	0	0	0	0	0	0
25 mn	0	0	0	0	0	0
28 mn	0	0	0	0	0	0
30 mn	0	0	0	0	0	0
33 mn	0	0	0	1	1	0
35 mn	0	0	1	1	2	0
38 mn	0	1	1	3	5	0
40 mn	1	2	2	3	8	0
43 mn	1	2	2	4	9	0
45 mn	1	3	3	4	11	0
48 mn	1	3	3	4	11	0
50 mn	1	4	3	4	12	0
53 mn	4	4	3	4	15	0
55 mn	4	5	3	4	16	0
58 mn	4	5	3	5	17	0
60 mn	4	6	4	6	20	0
80 mn					0	0

Nombre de Morts après 24 heures	1	1	0	1	3	0
Mortalité observée					3.09%	0.00%
Mortalité corrigée					3.09%	

TEST DE SENSIBILITE DES VECTEURS DE PALUD
INSECTICIDES

**Localities**	**Abengourou**
**Date**	**5/16/2016**
**Espèce vectrice**	***An. gambiae***
**Insecticide testé**	**Bendiocarb 0,1%**
**Replicat**	**4**

	TEMPERATURE		HUMIDITE
	AU DEBUT	A LA FIN	AU DEBUT
PERIODE D'EXPOSITION	24.9		52%
PERIODE D'OBSERVATION			

Témoin 2		Tube 1	Tube 2	Tube 3	Tube 4
25	Nombre de moustiques exposés	25	24	25	23
	Nombre de moustiques Kd après :				
0	5 mn	0	0	0	0
0	8 mn	0	0	0	0
0	10 mn	0	0	0	0
0	13 mn	0	0	0	0
0	15 mn	0	0	0	0
0	18 mn	0	0	0	0
0	20 mn	0	0	0	0
0	23 mn	0	0	0	0
0	25 mn	0	0	0	0
0	28 mn	0	0	0	0
0	30 mn	0	0	0	0
0	33 mn	0	0	0	0
0	35 mn	0	0	0	0
0	38 mn	0	0	0	0
0	40 mn	0	0	0	0
0	43 mn	0	0	0	0
0	45 mn	0	2	0	0
0	48 mn	0	3	0	0
0	50 mn	0	3	0	0
0	53 mn	0	3	2	0
0	55 mn	0	3	2	0
0	58 mn	4	4	2	1
0	60 mn	4	4	2	3
0	80 mn				

0
0.00%

Nombre de Morts après 24 heures	5	6	3	2
Mortalité observée				
Mortalité corrigée				

DISME AUX

RELATIVE
A LA FIN

Total Test	Témoin 1	Témoin 2
97	25	24
0	0	0
0	0	0
0	0	0
0	0	0
0	0	0
0	0	0
0	0	0
0	0	0
0	0	0
0	0	0
0	0	0
0	0	0
0	0	0
0	0	0
0	0	0
0	0	0
2	0	0
3	0	0
3	0	0
5	0	0
5	0	0
11	0	0
13	0	0
0	0	0

TEST DE SENSIBILITE

AUX

**Localities**	**Abengourou**
**Date**	**5/19/2016**
**Espèce vectrice**	***An. gambiae***
**Insecticide testé**	**DDT 4%**
**Replicat**	**4**

	TEMPERA
	AU DEBUT
PERIODE D'EXPOSITION	26°C
PERIODE D'OBSERVATION	

	Tube 1	Tube 2
Nombre de moustiques exposés	25	24
Nombre de moustiques Kd après :		
5 mn	0	0
8 mn	0	0
10 mn	0	0
13 mn	0	0
15 mn	0	0
18 mn	0	0
20 mn	0	0
23 mn	0	0
25 mn	0	0
28 mn	0	0
30 mn	0	0
33 mn	0	0
35 mn	0	0
38 mn	0	0
40 mn	0	0
43 mn	0	0
45 mn	0	0
48 mn	0	0
50 mn	0	0
53 mn	0	0
55 mn	0	0
58 mn	0	0
60 mn	0	0
80 mn		

16	0	0
16.49%	0.00%	0.00%
16.49%		

Nombre de Morts après 24 heures	0	0
Mortalité observée		
Mortalité corrigée		

DES VECTEURS DE PALUDISME
INSECTICIDES

Localities
Date
Espèce vec
Insecticide
Replicat

ATURE	HUMIDITE RELATIVE	
A LA FIN	AU DEBUT	A LA FIN
25,7°C	63%	65%

PERIODE D'EX
PERIODE D'OB

Tube 3	Tube 4	Total Test	Témoin 1	Témoin 2	
25	25	99	25	25	Nombre de moustiques exposés
					Nombre de moustiques Kd après :
0	0	0	0	0	5 mn
0	0	0	0	0	8 mn
0	0	0	0	0	10 mn
0	0	0	0	0	13 mn
0	0	0	0	0	15 mn
0	0	0	0	0	18 mn
0	0	0	0	0	20 mn
0	0	0	0	0	23 mn
0	0	0	0	0	25 mn
0	0	0	0	0	28 mn
0	0	0	0	0	30 mn
0	0	0	0	0	33 mn
0	0	0	0	0	35 mn
0	0	0	0	0	38 mn
0	0	0	0	0	40 mn
0	0	0	0	0	43 mn
0	0	0	0	0	45 mn
0	0	0	0	0	48 mn
0	0	0	0	0	50 mn
0	0	0	0	0	53 mn
0	0	0	0	0	55 mn
0	0	0	0	0	58 mn
0	0	0	0	0	60 mn
		0	0	0	80 mn

0	0	0	0	0	Nombre de Morts après 24 heures
		0.00%	0.00%	0.00%	Mortalité observée
		0.00%			Mortalité corrigée

TEST DE SENSIBILITE DES VECTEURS DE PALUDISME AUX INSECTICIDES

	**Abengourou**
	**5/19/2016**
**ctrice**	***An. gambiae***
**testé**	**Malathion**
	**4**

	TEMPERATURE		HUMIDITE RELATIVE	
	AU DEBUT	A LA FIN	AU DEBUT	A LA FIN
POSITION	25,9°C	25,5°C	62%	69%
SERVATION				

Tube 1	Tube 2	Tube 3	Tube 4	Total Test	Témoin 1	Témoin 2
25	25	25	25	100	25	25
0	0	0	0	0	0	0
0	0	0	0	0	0	0
0	0	0	0	0	0	0
0	0	0	0	0	0	0
0	0	0	0	0	0	0
0	0	0	0	0	0	0
0	0	0	0	0	0	0
0	1	5	1	7	0	0
3	4	6	5	18	0	0
8	9	10	5	32	0	0
9	12	11	8	40	0	0
10	16	14	12	52	0	0
12	16	15	14	57	0	0
17	20	21	14	72	0	0
18	21	22	14	75	0	0
18	21	22	17	78	0	0
18	22	24	18	82	0	0
19	24	24	20	87	0	0
21	24	24	22	91	0	0
21	24	24	22	91	0	0
22	24	24	22	92	0	0
22	25	24	23	94	0	0
23	25	24	23	95	0	0
				0	0	0

25	25	25	25	100	0	1
				100.00%	0.00%	4.00%
				100.00%		
